# Dr. Horace M. Moody

**Published:** 1912-03

**Authors:** 


Dr. Horace M. Moody, a graduate of the Geneva N. Y. Medical College, 1866, and a Civil War veteran, died at his home in E. Smithfield, Pa., Jan. 18, 1912, aged 73.

